# Relationships between team characteristics and soldiers’ organizational commitment and well-being: the mediating role of psychological resilience

**DOI:** 10.3389/fpsyg.2024.1353793

**Published:** 2024-01-29

**Authors:** Rosita Kanapeckaitė, Dalia Bagdžiūnienė

**Affiliations:** Institute of Psychology, Vilnius University, Vilnius, Lithuania

**Keywords:** soldier, psychological resilience, team characteristics, commitment, well-being

## Abstract

**Background:**

Military operations call for a great deal of readiness and resilience on the part of the soldiers, once confronted with high-stress scenarios. Resilience, in this context, has to do with the ability to effectively cope with the adverse impacts of setbacks and the accompanying stressors, ensuring that overall performance and combat effectiveness remain unhampered. In the modern military context, it is relevant to examine more deeply the phenomenon of soldiers’ resilience, its importance in positive organizational and personal outcomes as well as the role of team factors for the improving of soldiers’ resilience. The study aimed to examine team-level factors that determine soldiers’ psychological resilience and to reveal the mediating role of resilience in the relationships between team factors and organizational commitment and well-being.

**Sample:**

A cross-sectional convenience sample included 422 soldiers on professional military duty in the Lithuanian Armed Forces.

**Methods:**

Data were collected using self-administered questionnaire. We applied structural equation modeling to assess the research models.

**Results:**

Team cohesion and colleague support proved to be reliable predictors of increased psychological resilience; individuals with higher resilience were more committed to the organization and experienced higher well-being; the results confirmed the hypotheses that soldier resilience has a mediating effect on the relationships between team characteristics and their commitment, and well-being.

**Conclusion:**

The findings help clarify the relationships between team characteristics, soldiers’ resilience, commitment and well-being. They may be useful for improving soldier resilience through team cohesion, mutual support, cooperation, and for integrating team-building interventions into military resilience training programs.

## 1 Introduction

The Lithuanian Armed Forces as an organization continually updates its technology, weaponry, and various operational strategies. However, there is a significant gap in research related to the importance of individuals and their professional and personal competencies within the organization. When studying resilience, it is beneficial to apply an approach specific to the context (Stokes et al., 2019). Organizational resilience reflects the organization’s “ability to survive and potentially thrive during crisis” (McManus et al., 2008). Research on individual psychological resilience in a professional setting is still in its infancy. The issue of psychological resilience among military personnel is complex and multifaceted, requiring comprehensive scientific research.

Literature reveals that psychologically resilient soldiers are better equipped to lead and assist their colleagues. They can inspire their subordinates to develop similar psychological skills and attitudes, thereby fostering a culture of resilience within the military. Continuous improvement and adaptability are possible only in military organizations that actively seek to create an environment for enhancing and supporting psychological resilience. Research on psychological resilience among soldiers is dominated by clinical psychology contexts related to health impairment or PTSD prevention, while there is a lack of research related to the daily context of the profession and to the difficulties arising from everyday life. Psychological resilience is of particular importance in the military, in that service members often face various hardships, stressors, and traumas during their service. Recent conflicts, for instance, the war in Ukraine, have increased the need for research on psychological resilience and related phenomena. Technological advancements and the dynamic security environment contribute to constant changes within military organizations, placing pressure on soldiers and leaders to adapt and meet evolving challenges. Understanding what influences psychological resilience in soldiers can assist both soldiers and leaders in developing strategies for coping with stressful situations, thereby enhancing soldier commitment to the organization and their overall well-being.

Recently, researchers have been exploring resilience in civil applications to a greater extent. Research indicates that resilience is not limited to crisis management; it is a long-term capability that helps maintain physical and mental equilibrium in daily activities, and it can be strengthened and nurtured (Kuntz et al., 2016; Yost, 2016). Psychological resilience is a dynamic process that depends on the surrounding system, where various factors continuously interact (Masten, 2013). In Lithuania, psychological resilience in the context of work or military service has not been extensively studied. Resilience in the workplace has been investigated in various industries and professions, for example, general business organizations (Shin et al., 2012), healthcare (Gabriel et al., 2011), and the military (Lee et al., 2013). The majority of workplace resilience research focuses on the individual level (King et al., 2016). Conceptualizations of resilience at the individual level can be found in the works of Vanhove et al. (2015), Britt et al. (2016), Kossek and Perrigino (2016).

There is a paucity of research examining the specific characteristics of military organizations, with most studies being focused on healthcare workers and educators. Further exploration is needed to better understand the phenomenon of psychological resilience, attract and retain psychologically resilient military personnel, and ensure adequate national defense.

Limited research suggests that psychological resilience in soldiers may be one of the factors that determine their commitment and well-being. It is, therefore, appropriate to use an ecological resilience approach to investigate the team factors that enhance psychological resilience in soldiers. The current study aimed to examine team-level factors that determine soldiers’ psychological resilience and to reveal the mediating role of resilience in the relationships between team factors and organizational commitment and well-being.

Our study examines the issue of psychological resilience in soldiers from several perspectives: we look at team factors as antecedents of psychological resilience, or in other words, the context in which the soldier operates; we investigate the relationship between soldiers’ psychological resilience, commitment and well-being; and, most importantly, we seek to establish the role of psychological resilience as a mediating variable in the relationships between team factors and organizational commitment and well-being.

## 2 Theoretical background and research hypotheses

### 2.1 Psychological resilience of soldiers

Resilience can be understood as a cultivated competence to act in the face of adversity and to reflect on the crisis or challenge and subsequently improve (Kuntz et al., 2016) in the military context. Military personnel are exposed to a wide range of challenging and traumatic events during training, exercises, and deployments in combat environments that can be detrimental to their health, well-being and performance; and military psychologists are interested in the factors associated with resilience to these experiences. In the literature, resilience is often understood as the ability to “bounce back” from stressful events. Resilience is defined and measured in a variety of ways (Meredith et al., 2011; Fikretoglu and McCreary, 2012). Luthans and Youssef (2007) defines resilience as the ability to adapt “in the face of significant risk or adversity.” Luthans linked resilience to better attitudes toward work/activity and better health outcomes (Snyder, 2002). An Army study on psychological capital (PsyCap) by Schaubroeck et al. (2011) found that in a sample of soldiers deployed in Iraq, higher levels of PsyCap were associated with lower stress appraisal, and the protective effect of PsyCap was stronger for soldiers serving in units with higher levels of combat stress. Research on soldiers recognizes that personality can change throughout adulthood. Fikretoglu and McCreary (2012) defines resilience as a positive adaptation to significant adversity, with resilience being a response to stressful circumstances (e.g., demonstrating positive adaptation). When examining resilience in soldiers, researchers highlight that individuals in the military experience a range of traumatic events during combat training, missions and are constantly exposed to adverse environments as well as low-level stressors (e.g., living in difficult conditions, away from family) (Adler et al., 2011). It is widely acknowledged that psychological resilience is essential to cope with the cognitive, emotional and social stressors associated with the impact of war (Nindl et al., 2018). Psychological resilience is central to military preparedness as it plays an important role in dealing with physiological stressors; moreover, a soldier under psychological stress (i.e., a soldier who is unable to cope with psychological stressors) will not be able to carry out military operations well, however physiologically fit he or she may be (Nindl et al., 2018). Resilience and mental health are interrelated; it demonstrates that military personnel can effectively reduce negative psychological symptoms by improving their resilience level and adopting mature coping styles under stressful situations (Cao et al., 2023).

Contemporary scholarly discourse, as elucidated by Okojie et al. (2023), has advanced the understanding of employee resilience, reorienting the paradigm from a focus on intrinsic coping mechanisms in response to stress to a more contextual examination of resilience manifestation within daily occupational settings (Kuntz et al., 2016). This construct of employee resilience is theorized as a dispositional attribute, instrumental in catalyzing psychological processes that facilitate an individual’s recuperation from strenuous, traumatic, or catastrophic occupational experiences (Kuntz et al., 2016). Furthermore, this research posits employee resilience as a dynamic and malleable capacity within the realm of organizational studies, suggesting that it is not merely an innate trait but a developable faculty. The interplay between individual capacities and the occupational milieu plays a pivotal role in enabling employees to surmount professional obstacles (Kuntz et al., 2016). In light of this, the importance of organizational strategies aimed at nurturing employee resilience and fostering workplace engagement has become increasingly salient (Okojie et al., 2023). The dearth of research specifically addressing the unique daily stresses and challenges encountered by military personnel is a noteworthy gap in the current academic discourse. This lacuna is particularly significant, given the intrinsic characteristics of military service which inherently differ from civilian occupational contexts. In summary, the absence of focused research on the daily challenges faced by soldiers, considering the singular nature of military service, represents a critical gap in the broader field of stress and resilience studies.

### 2.2 Team characteristics and psychological resilience

Military service is fundamentally distinct from other forms of occupation due to its inherent emphasis on collective operation, typically within small unit structures. This characteristic positions military service as a prime subject for studies in team dynamics, situating it within the broader purview of environmental or contextual research in organizational psychology. We explored two team characteristics in this study – team cohesion and colleagues support.

#### 2.2.1 Team cohesion and soldier resilience

Team cohesion is a dynamic process that reflects the tendency of a team to stick together and remain united in pursuit of its goals (Kirke, 2010). A team is identified by its significant autonomy and capability to execute tasks necessitating member interdependence and role distribution (Rasmussen and Jeppesen, 2006). Participation in a team yields various beneficial outcomes such as commitment, job satisfaction, safe behavior, and effective performance (Rasmussen and Jeppesen, 2006). Teamwork holds a critical place in military operations. In contemporary warfare, the emphasis is on the deployment of smaller, highly efficient units. This approach is favored because it allows for rapid, adaptable and unpredictable actions, key attributes for successful military maneuvers (Society for Military Psychology, 2007). Unit cohesion has been found to have a strong positive relation with physical and psychological outcomes of military personnel (Williams et al., 2016). Research also has demonstrated the relation between unit cohesion and organizational outcomes, including perceptions of individual readiness and unit readiness (Griffith, 2002). Increased cohesion showed increased resilience, confidence, and managing react (Williams et al., 2016). Unit cohesion plays a key role in the psychological health of new soldiers, and positive social climates in operational units play a protective role with respect to the outcomes of well-being (Bliese and Britt, 2001). Cohesion is defined as the ability to establish trust and teamwork through members’ bonds (Ha and Jue, 2022). Because cohesion closely relates to work performance and adaptation to military life, it is necessary to pinpoint several methods for improving cohesion among soldiers (Ha and Jue, 2022). In challenging environments, reliable, and sustainable team performance and well-being is only possible when the team is resilient (Alliger et al., 2015). In military settings, team members often collaborate tightly, sharing knowledge, and striving toward common objectives when assigned a task. It is essential for team members, each with unique roles and duties, to cooperate effectively and adapt rapidly in order to accomplish shared goals, as highlighted by Lee et al. (2013). Moreover, a resourceful work environment – characterized by the availability of necessary tools, information, and support – empowers employees to perform their tasks more efficiently and effectively. It enables them to navigate challenges and leverage opportunities, thereby aligning their individual performances with broader organizational goals.

#### 2.2.2 Colleague support and soldier resilience

Support from colleagues has to do with a belief that colleagues are concerned about an individual’s well-being and notice their contribution to the overall performance (Ladd and Henry, 2000). Kox et al. (2022) showed that support is a decisive factor in achieving team goals, with higher levels of colleague support achieved under uncertainty. In uncertain, risky, and vulnerable situations found in the military, the role of colleague support in facilitating cooperation becomes particularly crucial. Support plays a pivotal role in team collaboration, significantly contributing to the attainment of collective objectives. It fosters self-assurance and a sense of security within the group and is instrumental in enabling team members to predict each other’s actions in scenarios requiring swift decision-making, as observed by Kox et al. (2022). Researchers identified the following determinants for resilience in the workplace (assumptions of resilience at the team level): emotions; collective positive emotions such as shared enthusiasm, optimism, comfort or relaxation, tend to increase resilience at the team level; and interpersonal processes (Stephens et al., 2013) wherein the ability to experience a range of emotions in teams was positively related to team resilience, and mediated the effects of intra-team trust on team resilience. Sharing negative emotions helped teams resolve their members’ problems, whilst sharing the positive ones helped them recover from difficulties (Stephens et al., 2013). Previous studies showed that the team members’ support can predict team performance. In many operational Army situations, teams of people are bystanders in the execution of tasks, which can lead to a breach of trust due to unfamiliarity, hence undermining cooperation (Kox et al., 2022). The impact of colleague support on employee well-being and performance is multifaceted. Perceived support from colleagues is often associated with enhanced team cohesion and a more collaborative work environment, fostering a culture of mutual respect and shared responsibility.

Effective social support, a key aspect of unit cohesion, has been linked to lessening the impact of traumatic stress and depressive symptoms among US veterans (Jones et al., 2012). This sense of cohesion likely plays a direct role in mental health by encouraging colleague support. It was noted that many personnel would prefer to turn to their fellow unit members for assistance with personal or emotional issues (Jones et al., 2012). Empirical studies in organizational psychology suggest that the quality of interpersonal interactions and the availability of support systems within a work setting are the crucial determinants for employee performance. This, in turn, fosters a sense of belonging and commitment among employees, which is pivotal for their psychological well-being and productivity. Research in this domain extends beyond the examination of individual behaviors to encompass the interactional patterns, shared norms, and collective decision-making processes that define the functionality of these units. The environmental or contextual approach in team research here acknowledges the interplay between individual soldiers and the overarching military system, including its hierarchical structure, cultural norms and operational demands. In summary, support from colleagues, encompassing both emotional empathy and recognition of contributions, plays a critical role in shaping an individual’s workplace experience. It not only bolsters personal well-being and motivation but also contributes to the development of a positive and productive organizational culture.

### 2.3 Psychological resilience and commitment to the organization

Commitment to the organization is an individual’s connection to the organization, manifested by his/her involvement in the organization’s activities, acceptance of the organization’s values and goals, willingness to remain a member (Meyer et al., 1993). Organizational researchers (e.g., Miller and Lee, 2001; Meyer et al., 2002) have long been interested in the topic of employee commitment to the organization. Two main approaches can be found in the literature: behavioral, sometimes described as exchange (Becker, 1960), and psychological, also known as attitudinal. Some authors identify a third approach, an integrative approach, which encompasses the first two (Cohen, 2006). Commitment to the organization refers to the employee’s emotional attachment, loyalty, and willingness to contribute to the success of the company. Commitment to the organization is often associated with job satisfaction, engagement and the intention to stay with the organization. The following factors contribute to commitment to the organization: job satisfaction, trust in management, alignment with the organization’s values and culture, fairness and justice in the workplace, and opportunities for personal and professional development. The lack of organizational commitment among soldiers has a negative impact on their productivity, which contributes significantly to their early exit from the armed forces and career change after receiving a full universal education. Team factors, resilience, and the relationship between commitment have been little studied by researchers. No research on these constructs has been carried out in Lithuania with professional soldiers.

### 2.4 Psychological resilience and well-being

Well-being is defined as a phenomenon that encompasses the positive and negative evaluations that an individual give to his or her life as a whole (Diener, 2006). Organizational researchers (e.g., Miller and Lee, 2001; Meyer et al., 2002) have long been interested in the topic of employee commitment to Two HRM concepts can improve employee and organizational performance, namely resilience and well-being (Merdiaty et al., 2021). Resilience and well-being have implications for different organizational arrangements, which depends on organizational management to provide employees with the needs they require to improve their performance (Merdiaty et al., 2021). Internal and external factors make resilience and well-being highly attractive to every organization, and the challenge of HRM is to create a balance to achieve the right level of resilience and well-being so that employees and organizations can work together to develop their creativity and productivity (Merdiaty et al., 2021). In resilience research, there is empirical evidence of positive relationships between organizational citizenship and corporate commitment to the organization (Merdiaty et al., 2021).

Some studies have shown that well-being has a positive impact on two forms of personal resilience: the worker’s ability to cope with stress (personal resilience) and resilient behavior in the workplace (employee resilience); apparently, there is an interesting relationship between the two concepts: whether employees with good well-being in the workplace can increase employee resilience, and vice versa (Merdiaty et al., 2021). Understanding the nature of the link between employee and personal resilience links resilience and well-being through positive and emotional affect to suggest that the two constructs are distinct but reciprocally related (Merdiaty et al., 2021). The gap between resilience and well-being varies considerably across organizational contexts and can be influenced by the state of the country, gender, politics, finance, leadership, organizational form and age, making it challenging to conceptualize appropriate interventions to improve performance (Merdiaty et al., 2021).

Resilience is promoted as an essential aspect of development in an uncertain world full of disruptions and surprises. However, these terms often remain ambiguous when applied, and it is not clear which term, that is, well-being or resilience, is used in different organizations (Chaigneau et al., 2022). Well-being is increasingly understood as a multidimensional concept consisting of objective indicators (what people have achieved or are able to achieve) and subjective measures (how they assess their situation). Well-being is not limitless and looks at how people create well-being in an environment of limited resources (Chaigneau et al., 2022). Resilience is also a multidimensional construct and is often defined as the ability of a system to withstand disturbances while maintaining its structure and functionality (Chaigneau et al., 2022). Both of these concepts are complex and are increasingly understood as dynamic and socially contingent. Taking into account the needs, values and contexts of specific contexts in their practical application is essential to ensure the measurement of well-being and resilience indicators (Chaigneau et al., 2022). Recent literature on well-being suggests that material, relational and subjective domains of well-being influence the person’s resilience and ability to adapt and cope with stressors and shocks (Chaigneau et al., 2022).

Relationships between individuals, communities, and organizations can help build resilience to change by providing social support and access to knowledge and resources (Chaigneau et al., 2022). It can be concluded that all dimensions of well-being can be the sources of resilience, as they influence adaptive capacity and in turn the potential for well-being improvement through adaptation (Chaigneau et al., 2022). Earlier research has demonstrated that individuals with resilience are able to sustain their physical and mental health by not only mitigating the detrimental effects of challenging periods but also by enhancing their psychological well-being (Connor and Davidson, 2003).

### 2.5 Mediating role of soldiers’ psychological resilience on commitment and well-being

From a social-ecological point of view, the field, or living space, can be understood as the social-ecological system that surrounds a human being – that is, an individual, a group, a community, an institution or a society. Field theory itself corresponds to many of the basic principles of socio-ecological research. In order to define the field or life space, Lewin first examined what he called psychological ecology, a life space within the designated area. It is the place where psychological, (subjective) and non-psychological (objective) factors intersect (Lewin, 1951). Ungar et al.’s (2013) research suggests that an ecological perspective helps us understand how people can develop resilience in a complex and changing world. Emphasizing the importance of connections to the social and physical environment, his work highlights the role of wider social and environmental factors in promoting resilience, rather than focusing solely on individual traits or characteristics.

Different models of resilience focus on the interaction between the individual and the resilience environment, where individuals mobilize personal and social resources in response to stressful situations to protect themselves from risk (Mak et al., 2011).

Organizational commitment refers to an individual’s psychological attachment to an organization, characterized by the strong identification with and the desire to maintain membership in the organization to support its goals (Vakola and Nikolaou, 2005). Research has been conducted on the relationship between employee burnout, organizational commitment, and the intention to leave a job in South Korean newspaper companies. It was discovered that employees experiencing burnout showed reduced organizational commitment and heightened intention to leave (Jung and Kim, 2012). Additionally, Son et al. (2022) observed that in healthcare workers during the COVID-19 pandemic, resilience plays a mediating role between organizational commitment, anxiety responses, and their quality of life. In the realms of resilience, human resource management, and fostering employee dedication to the organization, it becomes vital to enhance resilience and investigate its connections with other organizational elements, especially in more complex professional settings.

While few empirical studies directly examined the links between resilience and well-being, Mak et al. (2011) explored a mediating role of the positive cognitive triad (self-esteem, positive worldview, and hope) between resilience traits and well-being (Mak et al., 2011). In order to understand how students adapt to the daily stresses associated with student life, a positive thinking model was tested that may explain interconnectedness between resilience and well-being (Mak et al., 2011). Resilience was selected as a key construct in positive psychology and is believed to play an important role in promoting human well-being (Mak et al., 2011). Resilience was found to be related to the individual’s general adjustment, job performance, social functioning, physical and social psychological health (Mak et al., 2011).

Resilience research has so far paid insufficient attention to the multifaceted aspects of resilience and occupational context (Liu et al., 2019). In the context of resilience, well-being and human resource management, it is proposed to study resilience from an interdisciplinary perspective related to different occupational contexts. Understanding the contexts can improve the overall understanding of the importance of the phenomenon and how resilience can be developed from an organizational perspective (Liu et al., 2019). Increasing resilience in an organization can serve as one of the factors for positive change in organizational behavior research so that psychology focuses not only on the worst things that happen in life and fixing them, but also on creating a positive environment (Liu et al., 2019). Intuitively, resilience refers to recovery, as both individuals and organizations are exposed to stressful situations throughout their lifecycle (Liu et al., 2019). Thus, resilience can be an important intellectual concept to understand the differences in the behavior of organizational actors when they bounce back (Liu et al., 2019). The role of occupational context may have implications for theoretical developments in resilience research (Kossek and Perrigino, 2016). In organizational research, resilience can be understood as the skill and ability to be resilient in the face of overwhelming stress and change (Coutu, 2002).

From a dynamic perspective, resilience as a capacity can be increased and manifested in a dynamic process in response to traumatic events (Liu et al., 2019). Risk is a necessary component in the contexts studied. Risk is prevalent across domains and occupations, and effective risk management requires resilience (Liu et al., 2019). Different approaches to resilience are complementary since resilience has a multidimensional nature.

Recent research has identified the importance of resilience for community-level phenomena, such as resilience in entrepreneurial ecosystems (Roundy et al., 2017), while in modern society resilience is required in a wide range of organizational contexts. And examining resilience from an interdisciplinary perspective can help reveal new insights (Liu et al., 2019).

Different organizational environments also bring a critical perspective to the issue of levels of analysis when examining the antecedents, processes and consequences of resilience (Liu et al., 2019). Most existing resilience research stemming from positive psychology or positive organizational behavior tends to focus on the individual level (Liu et al., 2019). For example, sports players need to correct mistakes, put them aside and recover quickly (Liu et al., 2019). Entrepreneurs face adverse situations, high uncertainty, stressful events, and challenging circumstances and need resilience to achieve entrepreneurial performance (Bullough et al., 2014).

Increasingly, research is beginning to change the level of analysis in ways that have recognized the importance of team resilience in sporting activities (Morgan et al., 2013) and military training studies (Seligman, 2011). Moving to the organizational level, previous research has identified the importance of resilience in community-level phenomena such as entrepreneurial ecosystems (Roundy et al., 2017).

The Chinese financial services industry was investigated with a sample of 2040 banking employees. This study found that labor resources can positively affect resilience and subsequent employee engagement (Liu et al., 2019).

Branicki, Steyer, and Sullivan-Taylor sought to uncover microprocesses involved in building resilient organizations. The study interviewed 137 resilient managers from the United Kingdom and France. The authors juxtaposed everyday “business as usual” and extreme events as two scenarios to explore the implications for individual and organizational resilience. This study shows that microprocesses have important implications for resilience at both individual and organizational levels (Liu et al., 2019).

As resilience can be difficult if not impossible to develop, it may be easier for organizations to recruit people with high resilience levels. Shin et al. (2012) emphasize theories of resilience, suggesting that in response to demanding and stressful environments, individuals seek psychological or material resources to protect them from the effects of such stressors. Therefore, Shin et al. (2012) argue that organizations should undertake interventions to strengthen the individual resources of existing employees prior to any change in order to reduce the stress experienced during organizational change and enhance commitment to the organization (Tonkin et al., 2018).

Previous studies have indicated that resilience, a key human attribute, plays a significant role in influencing subjective well-being or happiness (Li et al., 2014; Tan et al., 2021). Individuals who exhibit resilience tend to show greater perseverance in challenging situations, handle daily hardships more effectively, and possess a stronger ability to deal with life stressors (Mandleco, 2000; Shreffler et al., 2021). People possessing greater resilience are capable of preserving their physical and mental health, as they are able to mitigate the adverse impacts of challenging circumstances (Connor and Davidson, 2003).

**Figure 1:**
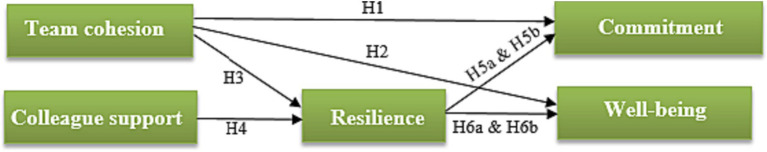
Theoretical model designed to characterize direct and indirect effects of three components team cohesion (TEM), colleague support (SUP), soldiers’ resilience (SREM): direct effects of team cohesion (TEM) to commitment (COM) are specified by hypothesis H1, to well-being (WLB) by H2, and to resilience (SREM) by H3; direct effects of colleagues’ support (SUP) to soldiers’ resilience (SREM) by H4; the indirect effects of team cohesion (TEM) (hypothesis H5a) and colleagues’ support (SUP) (hypothesis H5b) to commitment (COM) through soldiers’ resilience (SREM); and hypotheses H6a & H6b are specified to test the indirect effects of team cohesion (TEM, H6a) and colleagues’ support (SUP, H6b) to well-being (WLB) through soldiers’ resilience (SREM).

Expanding upon prior investigations, including Ungar et al.’s (2013) ecological viewpoint, this research posits that individuals can cultivate resilience amidst the complexities of a dynamic world. Emphasizing the significance of interactions with social and physical surroundings, this study delves into how social and environmental elements contribute to the formation of resilience, transcending mere individual attributes. The primary objective is to explore the interplay between team characteristics, soldiers’ psychological resilience, commitment and well-being. Crucially, this research seeks to understand the mediating function of psychological resilience in the relationship between team factors and soldier commitment and well-being. The hypotheses are visually presented in Figure 1.

## 3 Research hypotheses

The theorized direct and indirect links among study constructs were then tested: examining how team cohesion directly affects commitment and resilience in soldiers as well as their well-being; assessing a direct pathway between colleague support and soldier resilience; and evaluating indirect effects between all analyzed constructs.

*H1*: Team cohesion directly affects soldier commitment: soldiers who value team cohesion more highly are more committed to the organization.

*H2*: Team cohesion directly affects soldier well-being: soldiers who value team cohesion more highly also value their own well-being.

*H3*: Team cohesion directly affects soldier resilience: soldiers who value team cohesion higher are more psychologically resilient.

*H4*: Colleague support directly affects soldier resilience: soldiers who value colleague support higher are more psychologically resilient.

*H5a*: Soldier resilience positively mediates the relationship between team cohesion and commitment: team cohesion strengthens psychological resilience in soldiers, which increases their commitment to the organization.

*H5b*: Soldier resilience positively mediates the relationship between colleague support and commitment: colleague support strengthens psychological resilience in soldiers, which increases their commitment to the organization.

*H6a*: Soldier resilience positively mediates the relationship between team cohesion and soldier well-being: team cohesion strengthens psychological resilience in soldiers, which increases their well-being.

*H6b*: Soldier resilience positively mediates the relationship between colleague support and soldier well-being: colleague support strengthens psychological resilience in soldiers, which increases their well-being.

## 4 Research methodology

### 4.1 Research sample

This study used a random sampling method. Four-hundred twenty-two Lithuanian professional military service personnel participated in the study. The participants’ socio-demographic characteristics were as follows: 380 (90.0%) males and 39 (9.2%) females; three individuals did not indicate their gender. Participants ranged in age from 19 to 58 years with a mean age of 34.41 years (SD = 8.94). One-hundred fifty-five (36.7%) managers and 235 (55.7%) professionals took part in the survey: 28 (6.6%) respondents answered “other” to the question on job title, and four did not specify it at all. Two-hundred and one (47.6%) respondent had been working for up to 10 years, 104 (24.6%) for 10–20 years, 115 (27.3%) for more than 20 years, and two respondents did not indicate their length of service. The majority of participants, that is, 194 (46.0%), have a Bachelor’s or Master’s degree; 55 (13.0%) have a college degree, 51 (12.1%) – a vocational degree, and 119 (28.2%) – a secondary education. Two respondents did not indicate their educational background. An online self-completion questionnaire was used to collect the data. The questionnaire was not publicly available and was only open to soldiers who had received information about the study and an invitation to respond. The information and the invitation were distributed through the commanders of the army units. In the cover letter, we introduced the purpose of the study and provided instructions for completing the questionnaire. Participants were informed that the study was conducted in accordance with the ethical requirements of research, that the participants’ responses would be analyzed in aggregate for scientific purposes only, and that the confidentiality of their responses was guaranteed. Participation was entirely voluntary, without any remuneration.

### 4.2 Measures

The questionnaire consisted of demographic questions on respondents’ age, gender, education, length of service, the force in which they serve, and scales to measure the study variables: team characteristics (team cohesion and colleague support), psychological resilience, commitment to the organization and well-being. The whole composite concept assessment are presented in Table 1.

**Table 1:** The whole composite concept assessment.

Model variables	Statements	λ	CA	CR	AVE
Commitment	COM1	0.670	0.900	0.920	0.562
	COM2	0.709			
	COM3	0.682			
	COM4	0.765			
	COM5	0.807			
	COM6	0.834			
	COM7	0.827			
	COM8	0.689			
	COM 9	0.741			
Well-being	WLB1	0.607	0.840	0.892	0.582
	WLB 2	0.810			
	WLB 3	0.854			
	WLB 4	0.822			
	WLB 5	0.649			
	WLB 6	0.800			
Colleague support	SUP1	0.859	0.839	0.906	0.762
	SUP2	0.902			
	SUP3	0.857			
Team cohesion	TEM1	0.747	0.883	0.917	0.649
	TEM 2	0.832			
	TEM 3	0.852			
	TEM 4	0.841			
	TEM 5	0.861			
	TEM 6	0.685			
Soldiers’ resilience	SREM1	0.712	0.872	0.903	0.510
	SREM 2	0.669			
	SREM 3	0.722			
	SREM 4	0.737			
	SREM 5	0.761			
	SREM 6	0.761			
	SREM 7	0.738			
	SREM 8	0.570			
	SREM 9	0.734			

λ, standardized factor loadings; CA, Cronbach alpha; CR, construct reliability; AVE, average variance extracted.

#### 4.2.1 Team cohesion

Perceived team cohesion was measured using the Perceived Cohesion Scale developed by Bollen and Hoyle (1990), which has been used in a number of studies to assess the perceived cohesion of a group or team. The authors state that the scale consists of “Perceived Belonging” and “Emotional Experiences” subscales (Bollen and Hoyle, 1990). Principal component factor analysis of the Lithuanian sample identified one factor explaining 64.94% of the variance (KMO = 0.813; Bartlett’s sphericity chi2 = 1630.84, *p* < 0.001). The weights of the statements in the factor range from 0.470 to 0.741. In the light of these data, we analyzed one generalized indicator of team cohesion, which was calculated as the average of the responses to the six statements (e.g., “The soldiers in my team have a lot in common”). Responses are scored on a Likert scale ranging from 1 (strongly disagree) to 5 (strongly agree).

#### 4.2.2 Colleague support

Perceived colleague support was measured by the three-statement scale presented in the Copenhagen Psychosocial Questionnaire.^1^ Sample statement: “How often are your colleagues willing to listen to your problems at work if you need it?” Full psychometric descriptions of the Copenhagen Questionnaire and the individual scales can be found in Burr et al. (2019) and Kristensen et al. (2005). Responses ranged from 1 point (never) to 5 points (always). Principal component factor analysis in the present sample identified one factor, which explained 76.21% of the data variance (KMO = 0.713; Bartlett’s sphericity chi2 = 522.83, *p* < 0.001). The weights of the statements in the factor range from 0.734 to 0.814.

#### 4.2.3 Psychological resilience

Psychological resilience in soldiers was measured using the Employee Resilience scale which consists of nine items (Näswall et al., 2019). It is an employee-centered measure of psychological resilience that empirically examines resilience at the employee level, enabling organizations to monitor employee psychological resilience and identify areas that contribute to the development of their resilience. Sample statement: “I learn from mistakes in the service and improve the way I do my job.” Responses are scored on a Likert scale from 1 point (strongly disagree) to 5 points (strongly agree). Principal component factor analysis in our sample identified one factor explaining 50.94% of the variance (KMO = 0.892; Bartlett’s sphericity chi2 = 1568.86, *p* < 0.001). The weights of the statements in the factor range from 0.570 to 0.761.

#### 4.2.4 Commitment

Organizational Commitment was measured by The Organizational Commitment Questionnaire (OCQ) (Commeiras and Fournier, 2001; Yousef, 2003). The scale consists of 9 statements such as “I tell my friends about my organization as a great organization to work for.” Responses are scored from 1 – strongly disagree to 5 – strongly agree. Principal component factor analysis identified one factor explaining 56.20% of the variance (KMO = 0.909; Bartlett’s sphericity chi2 = 1942.65, *p* < 0.001). The weights of the statements in the factor range from 0.670 to 0.834.

#### 4.2.5 Well-being

Well-being was measured by combining indicators of life satisfaction and feelings of happiness. Life satisfaction was measured on a five-statement scale developed by Diener et al. (2009) (e.g., “For the most part, my life is close to my ideal”). Happiness was measured with one additional statement, “I feel like a happy person” (Diener, 2000; Ervasti and Venetoklis, 2010; Carr and Chung, 2014). Responses were rated on a five-point Likert scale, with 1 point (strongly disagree) and 5 points (strongly agree). Principal component factor analysis identified one factor that explained 58.16% of the variance in the data (KMO = 0.861; Bartlett’s sphericity chi2 = 1063.17, *p* < 0.001). The weights of the statements in the factor range from 0.607 to 0.854.

### 4.3 Statistical analyses

Statistical analyses were conducted utilizing IBM SPSS Statistics 29v and SPSS AMOS 29v. The individual level of analysis was applied to collected demographic data and study constructs, which include team cohesion, colleague support as well as resilience, commitment and well-being in soldiers. Descriptive statistics were employed to assess the statistical means and standard deviations (M and ± SD) of the construct variables. Subsequently, the Pearson bivariate correlation procedure was utilized to examine the relationships between constructs involved in this study. To mitigate the impact of common method bias in the study, two distinct methods were employed. The first approach involved the development of instruments that emphasized the anonymity and confidentiality of responses. As a second approach, Harman’s single-factor test was utilized to examine the potential variance introduced by common method bias (Tehseen et al., 2017). Structural Equation Modelling (SEM) served as the analytical framework to assess the hypothesized model. Preceding the modeling phase, a factor analysis was conducted to evaluate identified latent constructs and variables. Subsequently, the modeling process continued with theoretical causal model path analysis, following the methodology outlined by Crowley and Fan (2013). SEM identified causal interactions among eight factors. The theorized direct and indirect links among study constructs were then tested: examining how team cohesion directly affects commitment, resilience and well-being in soldiers (hypotheses: H1-H3); assessing the direct pathway between colleague support and soldier resilience (hypothesis: H4); and evaluating the theorized indirect effects between all constructs (hypotheses: H5a & H5b and H6a & H6b). Consistent with the proposed theoretical model design, five variables were recognized, comprising three observed endogenous variables (soldier resilience, soldier commitment, soldier well-being), two observed exogenous variables (team cohesion and colleague support).

The hypothesized relationships among model constructs were rigorously examined using SPSS AMOS 29v, with coefficient weights chosen to evaluate the causal relations. In accordance with the recommendations of previous scholars (Smaliukienė et al., 2023), who advocate for a multipurpose methodology for assessing the adequacy of a theoretical model, the goodness of fit was assessed based on several criteria. The following criteria were employed to evaluate model fit: the probability statistic of *χ*^2^ likelihood ratio, the Tucker and Lewis Index (TLI), the Comparative Fit Index (CFI), and the Root Mean Square Error of Approximation (RMSEA) with related confidence intervals (CI). Only values exceeding 0.95 for the TLI and CFI indices (Hu and Bentler, 1999) and values below 0.08 for the RMSEA measure (Browne and Cudeck, 1992) were considered acceptable. Data analysis and model parameter estimation were executed using the full information maximum likelihood method (Maydeu-Olivares et al., 2010). A bootstrapping analysis with 5,000 iterations was conducted, and confidence recognition was set at 95% for bias-corrected confidence intervals (95% CI). Following the criteria outlined by Hair (2019) and Hayes and Scharkow (2013), the effects of indirect relationships were deemed statistically significant if zero was not included in the 95% bias-corrected CI.

## 5 Results

The scholarly literature pertaining to structural equation modeling consistently advocates a systematic two-step approach for the comprehensive evaluation of models incorporating latent variables, as delineated in studies spanning references (Kanapeckaitė et al., 2022). In adherence to this established methodology, our investigation comprised two pivotal phases: first, the examination of the adequacy and construct validity of our measurement model; second, the examination of structural models and associated hypotheses. Throughout both stages of analysis, we applied the maximum likelihood procedure, a widely endorsed statistical technique in the field of structural equation modeling. This particular approach ensures a rigorous assessment of the proposed models, contributing to the robustness and reliability of our research findings (Table 1).

### 5.1 Preliminary analyses for scale evaluations

In the initial stage of our analysis, we assessed the conceptual model for its adequacy. Descriptive statistical analysis was conducted at the individual level, and preliminary information on research variables was gathered. The normality of the data was deemed acceptable, as indicated by kurtosis and skewness measurements, with absolute values below 3 and 7, respectively—meeting established criteria (Wang et al., 2020). Additionally, a thorough check for multicollinearity showed no evidence thereof. All tolerance values exceeded the 0.20 threshold, signifying the absence of multicollinearity in the examined variables. This analysis ensures the independence of variables, reinforcing the integrity of subsequent analyses. Furthermore, we estimated the convergent and discriminant validity of the designed constructs, which involved examining correlations and assessing convergent and discriminant validity for all variables in the conceptual model. The results of this analysis are presented in Table 2.

**Table 2:** Descriptive statistics, correlations, and scales’ Cronbach’s alpha coefficients.

Model variables	Descriptive statistics		Correlations				
	M	± SD	COM	WLB	SUP	TEM	SRES
*Dependent variables*							
Commitment (COM)	3.780	0.661	(0.900)				
Well-being (WLB)	3.784	0.628	0.538^**^	(0.840)			
*Independent variables*							
Colleague support (SUP)	3.893	0.811	0.404^**^	0.331^**^	(0.839)		
Team Cohesion (TEM)	3.809	0.689	0.572^**^	0.384^**^	0.533^**^	(0.883)	
*Mediator*							
Soldier resilience (SREM)	4.002	0.509	0.515^**^	0.511^**^	0.460^**^	0.433^**^	(0.872)

Pearson’s correlation is significant at: **p < 0.01 level (2-tailed). M – mean; ± SD – standard deviation. Cronbach’s alpha coefficients are shown in parentheses on the diagonal.

The correlation coefficients presented in Table 2 showed that commitment is positive associated with soldier well-being (*r* = 0.538, *p* < 0.01), team cohesion (*r* = 0.572, *p* < 0.01) and colleague support (*r* = 0.404, *p* < 0.01). Commitment indicated positive and highly significant relationship with employee resilience (*r* = 0.515, *p* < 0.01). The employee resilience showed statistically significant relations with soldier well-being (*r* = 0.511, *p* < 0.01), colleague support (*r* = 0.460, *p* < 0.01) and team cohesion (*r* = 0.433, *p* < 0.01).

Subsequently, confirmatory factor analysis was rigorously conducted to assess the validity of the constructed model, which includes five distinct constructs. The results clearly demonstrated satisfactory convergent validity, meeting established criteria (Byrne, 2013; Bekesiene et al., 2023). Importantly, all factor loadings were statistically significant at the value of *p* <0.001 level, affirming the robustness of the model and providing a solid foundation for subsequent stages of analysis and interpretation.

### 5.2 Hypotheses testing results

Modeling analysis was conducted using IBM AMOS 29v software. Confirmatory factor analysis was employed to test the theorized links among constructs in specified models: Model 1 investigated how team cohesion influences commitment (H1); Model 2 evaluated the pathway between team cohesion and soldier well-being (H2); Model 3 verified the effect of team cohesion on soldier resilience (H3); and Model 4 tested the impact of social support on soldier resilience (H4). Finally, the indirect effects theorized by hypotheses H5a & H5b and hypotheses H6a & H6b between all constructs were assessed. The goodness-of-fit of the theorized model was evaluated based on test results designed to demonstrate the model fit.

#### 5.2.1 Evaluation of direct effects based on study hypotheses

Firstly, we examined the direct effects of team cohesion on soldier commitment (H1, Model 1), soldier well-being (Model 2), and soldier resilience (H3, Model 3). Additionally, we estimated the direct effect of social support on soldier resilience (Model 4).

Examination of the hypothesized direct effects within the study model was conducted through the application of structural equation modeling analysis. The obtained results indicated a commendable fit to the data (*χ*^2^ = 1.048 [df = 2, *p* = 0.59], CFI = 1.000; NFI = 0.999; TLI = 1.007; RMSEA = 0.000, 90% CI: 0.00–0.08; and PCLOSE = 0.819). Notably, the *χ*^2^ test of exact fit demonstrated statistical significance, and the Comparative Fit Index (CFI) surpassed the recommended threshold value of 0.90, as proposed by Hu and Bentler (1999) and Maydeu-Olivares and García-Forero (2010). Furthermore, the Root Mean Square Error of Approximation (RMSEA) for the close fit test was well below the established threshold of 0.08, as outlined by Hair (2019).

Moreover, the analysis revealed a substantial, positive and direct impact of team cohesion on soldier commitment (H1: β = 0.430, *p* < 0.001), soldier well-being (H2: β = 0.201, *p* < 0.001) and soldier resilience (H3: β = 0.262, *p* < 0.001). Consequently, the hypotheses positing the direct effects of team cohesion were substantiated for soldier commitment (H1), soldier well-being (H2) and soldier resilience (H3) (refer to Model 1, Table 3).

**Table 3:** The results of direct effects of hypothesized model evaluated by SEM analysis.

Evaluation		Coeff. β	S.E.	St. Coeff. β	C.R.	*p*
H1	*Model 1*					
	TEM → COM	0.412	0.040	0.430	10.393	***
		Coeff. β	S.E.	St. Coeff. β	C.R.	*p*
H2	*Model 2*					
	TEM → WLB	0.183	0.041	0.201	4.424	***
		Coeff. β	S.E.	St. Coeff. β	C.R.	*p*
H3	*Model 3*					
	TEM → SREM	0.193	0.037	0.262	5.282	***
		Coeff. β	S.E.	St. Coeff. β	C.R.	*p*
H4	*Model 4*					
	SUP → SREM		0.201	0.031	0.320	6.463

Commitment (COM), Well-being (WLB); Colleague support (SUP); Team cohesion (TEM); Soldier Resilience (SREM). Standardised coefficients, St. Coeff. β. Critical Ratio for regression weight, C.R.; Standard error of regression weight, S.E Significance at: ***p < 0.001 (2-tailed); 5,000 sample size for bootstrap was used.

Furthermore, the undertaken analysis facilitated the examination of the direct effects of colleague support on soldier resilience. The findings indicated that support exerts a substantial, positive, and direct impact on soldier resilience (H4: β = 0.320, *p* < 0.001). Consequently, the hypothesis H4 postulating the direct effect of colleague support on soldier resilience was validated. Complete details are provided in Table 3.

#### 5.2.2 Mediation effect of resilience

Consistent with the research methodology, the theorized indirect effects (hypotheses: H5a & H5b and H6a & H6b) were subjected to examination. Hypotheses H5a & H5b were formulated to investigate the mediating relationships involving soldier resilience as a mediator in the relationship between team cohesion and commitment. Additionally, hypotheses H6a & H6b explored the relationships between team cohesion and resilience and well-being among soldiers.

The analysis carried out showed that the designed model indicated good consistency with the collected data (*χ*^2^ = 1.048 [df = 2, p = 0.59], CFI = 1.000; NFI = 0.999; TLI = 1.007; RMSEA = 0.000, 90% CI: 0.00–0.08; and PCLOSE = 0.819). The detailed study results presented in Table 4 confirmed that team cohesion and colleague support was positively and significantly related to soldier commitment: team cohesion (H5a: β = 0.086, *p* < 0.001), and colleague support (H5b: β = 0.106, *p* < 0.001). Additionally, soldier resilience was positively related to soldier commitment (β = 0.330, *p* < 0.001) and soldier well-being (β = 0.424, *p* < 0.001). Approximately 42% of the variance in soldier commitment was accounted by the predictors (*R*^2^ = 0.416, Table 4) and 30% of the variance for soldier’ well-being (R^2^ = 0.294, Table 4).

**Table 4:** The effect of team cohesion and colleague support on soldier commitment and well-being by soldier resilience evaluated by using SEM analysis.

	Soldier resilience	Commitment				Well-being			
Variables	Direct	Direct	Indirect	95% CI		Direct	Indirect	95% CI	
	St. Estim. β	St. Estim. β	St. Estim. β	LLCI	ULCI	St. Estim. β	St. Estim. β	LLCI	ULCI
*Independent variables*									
Team cohesion	0.262***	0.430***	0.086***	0.051	0.133	0.201***	0.111***	0.064	0.166
Colleague support	0.320***		0.106***	0.063	0.160		0.136***	0.085	0.193
*Mediator*									
Soldier resilience		0.330***				0.424***			
Model assessment by *R*^2^	0.260	0.416				0.294			

St. Estim. β– standardized estimations. Significance at: *p < 0.05; **p < 0.01; ***p < 0.001 (2-tailed). *R*^2^ – squared multiple correlation. Lower limit of 95% CI, LLCI; Upper limit of 95% CI, ULCI; bootstrap sample size = 5,000.

Furthermore, the outcomes of the modeling regarding the indirect effects of team cohesion and colleague support on commitment and well-being via soldier resilience as a mediator were assessed using the bias-corrected percentile bootstrap approach with 5,000 bootstrap samples, estimated at a 95% confidence interval. The analysis established positive and statistically significant indirect relationships from team cohesion (standardized effect = 0.086, *p* < 0.001, 95% CI = [0.051, 0.133]) and colleague support (standardized effect = 0.106, *p* < 0.001, 95% CI = [0.063, 0.160]) to commitment (Table 4).

Moreover, the weighted indirect effect through soldier resilience on soldier well-being was affirmed: from team cohesion (H6a: standardized effect = 0.111, *p* < 0.001, 95% CI = [0.064, 0.166]) and colleague support (H6b: standardized effect = 0.136, *p* < 0.001, 95% CI = [0.085, 0.193]). Subsequent to the bootstrap test with a sample size of 5,000 and a 95% CI excluding zero, the significant indirect effects of team cohesion and colleague support on well-being via soldier resilience were revealed (Table 4).

While the modeling results indicated that the mediation of soldier resilience for soldier commitment can be characterized as “partial mediation” (Baron and Kenny, 1986) for team cohesion (β = 0.430, *p* < 0.001), and soldier well-being (β = 0.201, *p* < 0.001), a distinct situation emerged concerning soldier resilience mediation effects when assessing the impact of colleague support on commitment and well-being. Colleague support did not exhibit a direct link with commitment, but indirect effects (β = 0.106, *p* < 0.001 for commitment and β = 0.136, *p* < 0.001 for well-being) were identified as positive and significant. Accordingly, soldier resilience fully mediates the relationships between colleague support and commitment as well as well-being (Figure 1).

**Figure 2:**
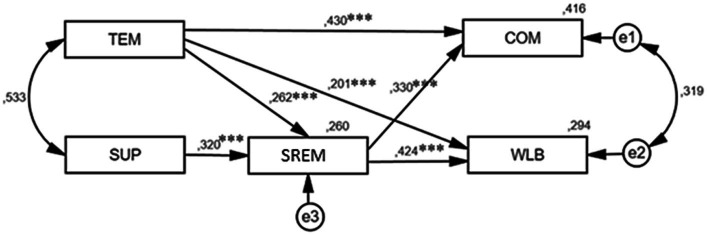
Graphical representation of structural modeling analysis results when mediation effect of soldier resilience is taken into account (*χ*^2^ = 1.048 [df = 2, *p* = 0.59], CFI = 1.000; NFI = 0.999; TLI = 1.007; RMSEA = 0.000, 90% CI: 0.00–0.08; and PCLOSE = 0.819). The standardized path coefficients are presented close to the arrows and significance indicator (***p < 0.001) is marked up.

Accordingly, the hypotheses H5a and H5b, asserting that “soldier resilience positively mediates the relationship between team cohesion and colleague support for soldier commitment” can be partially confirmed for team cohesion (H5a) and fully confirmed for colleague support (H5b). Similar situations arise with H6a & H6b, which posit that “soldier resilience positively mediates the relationship between team cohesion and colleague support for soldier well-being.” Resilience fully mediates colleague support (H6b) but only partially mediates team cohesion (H6a). The modeling results are visually depicted in Figure 2, and all estimates of the model are methodically detailed in Table 4.

## 6 Discussion

This research focused on evaluating direct and indirect relationships among the dimensions of team cohesion, colleague support, soldier resilience, commitment to the organization and well-being. Based on the ecological system theory (Krebs, 2009), an ecosystem is described as a sophisticated network of relationships involving developing individuals and their interactions with the environment (Liu et al., 2019). This theory underscores the reciprocal nature of the relationship between individuals and their environmental context.

To our knowledge, our study is one of the first to examine the selected factors in a sample of soldiers from the perspective of organizational psychology and human resources, whereas previous studies have mostly focused on soldiers’ health or its factors. The research suggests that team characteristics - team cohesion and colleague support – are important factors affecting psychological resilience among soldiers. According to the previous studies positive social relationships function as an important factor affecting mental resilience (Bartone et al., 2012). Previous studies mostly focused on the resilience role in preventing the post-traumatic stress disorder and coping styles (Zhao et al., 2020).

Results of the study show that team cohesion directly affects commitment in soldiers. There is a paucity of research examining the specific characteristics of military organizations. Teamwork holds a critical place in military operations. These results are consistent with earlier studies showing positive relationships between team cohesion and commitment. Participation in a team yields various beneficial outcomes, such as organizational commitment, job satisfaction, safe behavior, and effective performance (Rasmussen and Jeppesen, 2006).

Received results also propose that team cohesion directly affects soldier well-being, because cohesion closely relates not just to soldier performance but to their adaptation to military life, as well. Supportive relationships act as social buffers against work-related stressors, facilitating a more pleasant and intrinsically rewarding work experience. This, in turn, fosters a sense of belonging and commitment among soldiers, which is pivotal for their psychological well-being and productivity. Unit cohesion plays a key role in the psychological health of new soldiers, and positive social climates in operational units play a protective role with respect to well-being outcomes (Bliese and Britt, 2001).

Results of our study also propose that team cohesion directly affects soldier resilience. Team resilience is a shared construct that emerges through composition (Stephens et al., 2013). Stephens et al. (2013) conceptualize team resilience as an emergent state that describes the characteristics of a team, which are typically dynamic in nature and change depending on a team context, inputs, processes, and outcomes. This perspective emphasizes the team level of analysis and describes team resilience as a result of the interaction between contextual factors and team members.

Colleague support directly affects soldier resilience. In military settings, team members often collaborate tightly, sharing knowledge and striving toward common objectives when assigned a task. It is essential for team members, each with unique roles and duties, to cooperate effectively and adapt rapidly in order to accomplish shared goals, as highlighted by Lee et al. (2013). When an emotional regulation mode of self-comfort is carried out, the role of social support becomes greater, and a more positive coping method can be adopted. Supportive relationships act as social buffers against work-related stressors, facilitating a more pleasant and intrinsically rewarding work experience.

The current modeling analysis conducted confirmed the partly mediation of resilience between team cohesion and both dependent variables – commitment and well-being among soldiers.

This study has shown that resilience mediated the relationship between team cohesion and commitment to the organization. This finding explains that it is common in the military environment to operate in teams and small units, and that their members, individually and collectively, increase the soldier’s individual resilience, which in turn increases his/her commitment to the organization. A committed soldier is more effective in performing tasks and has a greater sense of satisfaction with service and pride in the organization to which he belongs. Soldiers with higher commitment demonstrate loyalty and have greater intrinsic motivation to perform, which can be valuable in certain roles and in leadership, as well.

Furthermore, this study confirmed that resilience fully mediated the dimension of colleague support and commitment. In the military context, colleagues play a particularly important role. As Ungar et al.’s (2013) research shows, social and environmental factors foster resilience, and resilience in turn fosters identification with the unit in which soldiers serve and their commitment. Supportive relationships promote a sense of belonging and commitment among soldiers. The ability to experience a range of emotions in teams is positively related to team resilience and mediated the effects of intra-team trust on team resilience. Sharing negative emotions helped teams resolve their members’ problems, while sharing positive emotions helped teams recover from difficulties (Stephens et al., 2013).

This study has shown that resilience mediated the relationship between team cohesion and well-being. Soldiers who experience higher levels of team cohesion demonstrate greater resilience, which in turn increases their well-being. Taking into account the needs, values and contexts of specific contexts in their practical application is essential to ensure the measurement of well-being and resilience indicators (Chaigneau et al., 2022). Exploring the phenomenon of resilience in the military context thus makes sense, as it can help professionals in the field of human resources management develop effective measures and programs for improving soldier resilience.

Furthermore, this study confirmed that resilience fully mediated the relationship between colleague support and soldier well-being. Overall, this is partly in line with previous research. In our case, the context chosen for the team factors reveals a new angle on resilience as a competency that is developed at work and leads to desirable outcomes. Earlier research has demonstrated that individuals with resilience are able to sustain their physical and mental health by not only mitigating the detrimental effects of challenging periods but also by enhancing their psychological well-being (Connor and Davidson, 2003).

## 7 Conclusion and implications

This research contributes to the existing body of knowledge on resilience by demonstrating that the beneficial impacts of military team dynamics on soldier commitment to the organization and well-being are mediated through resilience. Prior studies predominantly concentrated on aspects such as stress reduction and interventions related to health and the prevention of health disorders. Our study specifically reveals that team factors enhance soldier resilience, which subsequently leads to increased commitment and well-being among individuals. Therefore, our findings not only corroborate but also expand upon previous research that has established connections between resilience and various organizational factors. Different models of resilience focus on the interaction between the individual and the resilience environment, where individuals mobilize personal and social resources in response to stressful situations to protect themselves from risk (Mak et al., 2011). Ungar et al.’s (2013) study advocates for an ecological approach to comprehend how individuals cultivate resilience in a multifaceted and evolving global context. This perspective underscores the significance of an individual’s interaction with their social and physical environments. Ungar’s research illuminates what a crucial role broader social and environmental influences play in promoting resilience, moving beyond a narrow focus on personal traits or characteristics. Our study reveals that team cohesion and colleague support in the military context are important for soldier resilience. Therefore, when studying psychological resilience in soldiers, it is important to consider not only the factors of individual resilience but also the importance of their immediate social environment. Further research is needed to understand how soldier resilience and organizational resilience can be fostered through organizational interventions in order to determine to what extent such resilience indicators actually matter Tonkin et al. (2018).

From a practical point of view, the results of the study can help design interventions to increase resilience in soldiers and have a positive impact on their well-being and commitment to the organization.

## 8 Limitations

This research acknowledges certain constraints that must be considered when interpreting its findings. A primary limitation is the reliance on self-reporting instruments for evaluating team characteristics, resilience, commitment and well-being. In this context, geopolitical circumstances may have affected soldier well-being, potentially biasing self-assessments. Another constraint is the limited representation of female participants in the study, which may have implications for the comparative analysis of personal characteristics across gender groups. Additionally, it is important to recognize that team factors are not the sole predictors of soldier resilience. Resilience might be influenced by a multitude of elements, including but not limited to, training, leadership style and current service environment. Other influential factors such as educational background and leadership approach also play a significant role in shaping resilience, commitment, and well-being in soldiers.

Structural organization of military operations, predominantly executed through small, cohesive units, necessitates a high degree of interdependence and coordination among soldiers. This environment creates a unique socio-psychological context, wherein team dynamics, including communication, leadership, group cohesion, and conflict resolution, become critical factors influencing both individual and unit performance.

## Data availability statement

The raw data supporting the conclusions of this article will be made available by the authors, without undue reservation.

## Ethics statement

Before the start of the survey, the permission of the Ethics Committee of the Institute of Psychology of Vilnius University no. 74 (2022.01.06) was obtained.

## Author contributions

RK: Writing – original draft. DB: Writing – original draft.

## Footnotes

^1^https://www.copsoq-network.org/licence-guidelines-and-questionnaire/
